# Efficacy of nGVS to improve postural stability in people with bilateral vestibulopathy: A systematic review and meta-analysis

**DOI:** 10.3389/fnins.2022.1010239

**Published:** 2022-09-28

**Authors:** Ruth McLaren, Paul F. Smith, Rachael L. Taylor, Shobika Ravindran, Usman Rashid, Denise Taylor

**Affiliations:** ^1^Rehabilitation Innovation Centre, Health and Rehabilitation Research Institute, School of Clinical Science, Auckland University of Technology, Auckland, New Zealand; ^2^Department of Pharmacology and Toxicology, School of Biomedical Sciences, The Brain Health Research Centre, University of Otago, Dunedin, New Zealand; ^3^Eisdell Moore Centre for Hearing and Balance Research, University of Auckland, Auckland, New Zealand; ^4^Department of Physiology, University of Auckland, Auckland, New Zealand

**Keywords:** vestibular rehabilitation, balance, neuromodulation, noisy galvanic vestibular stimulation, nGVS, physical therapy

## Abstract

**Objective:**

Noisy galvanic vestibular stimulation (nGVS) has been used to boost vestibular afferent information to the central nervous system. This has the potential to improve postural control for people for whom vestibular signals are weak, such as in bilateral vestibulopathy (BVP). The aim of this systematic review and meta-analysis is to investigate the evidence for nGVS as a modality to improve postural control in people with BVP.

**Methods:**

A comprehensive systematic search was conducted of five databases up to July 2022 to find studies applying nGVS to people with BVP, with the aim of improving postural control. Two independent reviewers screened and identified eligible studies, completed a risk of bias evaluation (Cochrane) and extracted relevant data. The standardized mean difference (SMD) based on Hedges' g was calculated as a measure of effect size for the primary outcome measure that best identified postural control, and a forest plot generated.

**Results:**

Seven studies met the eligibility criteria, with five being suitable for meta-analysis. Meta-analysis revealed a moderate effect in favor of nGVS improving postural control during standing and walking [pooled SMD = 0.47 95% CI (0.25, 0.7)]. nGVS-mediated improvements in postural control were most evident in observations of reduced sway velocity when standing on a firm surface with eyes closed, and in the reduced variability of gait parameters, particularly those measuring lateral stability.

**Conclusions:**

Coincident nGVS in people with BVP improves postural control during standing and walking. This improvement appears to be context specific, in that vestibular augmentation is most effective in situations where visual inputs are limited, and where reliable context specific proprioceptive cues are available. Further research is warranted investigating additional circumstances in which nGVS improves postural control, including investigating the residual, and sustained effects of nGVS.

**Systematic review registration:**

https://www.crd.york.ac.uk/prospero/display_record.php?RecordID=342147, identifier: 342147.

## Introduction

### The vestibular system and bilateral vestibulopathy

The vestibular system is a closed system residing in the bony vault of the temporal bone and is extensively involved in the control of balance and eye movement. Consisting of the three semicircular canals and the two otolith organs (the utricle and saccule), the vestibular system relies on the inertial drag of endolymph and the static effects of gravity on its membranous labyrinth, to mechanically stimulate sensors that provide information to the brain about head position and movement of the head in space (Schubert and Minor, 2004). At a basic level it contributes to visual stability *via* the vestibulo-ocular reflexes and the maintenance of muscle tone, body and head posture and balance *via* the vestibulospinal reflexes (Schubert and Minor, 2004; Smith, 2017). See Schubert and Minor (2004) and Cullen (2019) for an extensive review of the physiological function of the vestibular system.

Bilateral vestibulopathy (BVP) is a diagnosis indicating that the neural signals that travel from the vestibular apparatus to the central nervous system are either absent or significantly reduced on both sides (Lucieer et al., 2020). There are multiple potential causes for BVP. Damage to the sensory organs of the vestibular system, the semicircular canals, utricle or saccule may occur in Meniere's disease, mechanical trauma, labyrinthitis or aminoglycoside toxicity (Kim and Kim, 2022) or the vestibular nerve may be impaired through neuritis, vestibular schwannoma or resection of the vestibular nerve (Kim and Kim, 2022). Around half of BVP cases are idiopathic, making the pathological process difficult to ascertain (Hain et al., 2018; Kim and Kim, 2022).

The reduced neural signals traveling from the vestibular apparatus to the central nervous system in people with BVP lead to imbalance, oscillopsia (visual blurring with head movement) and difficulty walking in darkness or over uneven surfaces (Ward et al., 2013; Lucieer et al., 2018, 2020). People also report deficits in spatial navigation, orientation (Schoberl et al., 2021), concentration, memory (Lucieer et al., 2018) and mood (Lucieer et al., 2020). As a result of these symptoms, the majority of patients report altering their behavior by avoiding activities, assigning more concentration to tasks, or completing tasks more slowly (Lucieer et al., 2020), and 35% of BVP patients perceive their vestibular loss as severely impacting their participation in daily activities (Dobbels et al., 2020).

Vestibular hair cells and neurons do not regenerate, and there is currently no established remedial medical treatment for BVP once damage has occurred (Guinand et al., 2012; Kim and Kim, 2022). To date, the gold standard treatment has been vestibular rehabilitation, which relies on central compensation and reweighting of other sensory inputs (Hall et al., 2016; Hain et al., 2018). However, vestibular rehabilitation has had modest and somewhat inconsistent effects (Herdman et al., 2015). An emerging treatment option is noisy galvanic vestibular stimulation (nGVS), which is a low-level noisy current that has been used successfully to facilitate both postural stability and vestibular ocular reflex activity (Stefani et al., 2020).

### Noisy galvanic vestibular stimulation

Noisy galvanic vestibular stimulation (nGVS) applied to the bilateral mastoid processes has been investigated over the past two decades as a treatment to improve balance and postural control (Iwasaki et al., 2014, 2018; Fujimoto et al., 2016, 2018; Wuehr et al., 2016a,b; Inukai et al., 2018a, 2020a,b,c; Ko et al., 2020; Chen et al., 2021; Nooristani et al., 2021). Delivered *via* electrodes placed bilaterally over the mastoid processes, nGVS is a zero mean, noisy galvanic current applied over a fixed bandwidth. While the exact mechanism of nGVS is unknown, the benefits have been observed primarily at low amplitudes (Iwasaki et al., 2014), leading to the assumption nGVS amplifies weak vestibular afferent signals *via* stochastic resonance (Wuehr et al., 2018). Stochastic resonance is a phenomenon during which a non-linear system that is operating at a subthreshold level is boosted by adding noise, bringing the system up to threshold (Galvan-Garza et al., 2018). In the vestibular system this increases the likelihood of neural firing and facilitates the restoration of missing sensory information to the central nervous system (Moss et al., 2004). When a weak vestibular signal is boosted by the addition of a small nGVS signal, the performance of the vestibular system is enhanced, and balance improves (Dlugaiczyk et al., 2019). The aim of this systematic review and meta-analysis is to evaluate the efficacy of nGVS as a modality to improve postural control in people with BVP.

## Methods

A literature search was undertaken using EBSCO (CINAHL plus, MEDLINE, SPORTDiscus), Scopus, Ovid (AMED) and Medline (PubMed). We used the search terms “bilateral vestibulopathy OR BVP OR bilateral vestibular weakness OR bilateral vestibular OR bilateral vestibular hypofunction OR BVH”; “AND nGVS OR noisy galvanic stimulation OR noisy vestibular stimulation OR galvanic vestibular stimulation OR GVS OR SVS OR stochastic vestibular stimulation.” Additional studies were identified by hand-searching the reference lists of key articles. Studies were restricted to peer reviewed journals with full text available in English, no limit was placed on the publication date or study design.

Two independent reviewers (RM, SR) screened titles and abstracts, and where necessary, the full text for eligibility according to the criteria in Table 1. In the case of any uncertainty, a third reviewer (DT) was consulted until consensus was achieved. The literature search was last performed on July 13, 2022.

**Table 1:** Inclusion and exclusion criteria.

	**Inclusion criteria**	**Exclusion criteria**
Population	Human	Animal studies
	Adults aged over 18 years	
	Diagnosed with BVP	
Intervention	Bipolar noisy galvanic current applied over the mastoid processes	Stimulation with the goal of perturbing balance or gait
Control	No nGVS or sham nGVS	
Outcomes	Physiological gait or balance measures	
Trial design	Original primary data	Review articles
	Pre/post experimental designs, crossover designs, randomized controlled trials	Studies using secondary data
Data	Full text available	
	Peer reviewed journal	
	English	

Data were extracted directly from the text, tables, supplementary files and where necessary from graphs using online software (https://apps.automeris.io/wpd/). Data extracted included, study design, sample size, participant characteristics, primary and secondary outcome measurements related to postural control, and study findings. Normalized ratio or percentage were calculated as appropriate.

### Risk of bias and trial quality assessment

The Cochrane risk of bias assessment (RoB2) for a randomized crossover design was applied to studies in the meta-analysis (Higgins et al., 2011). Data were extracted from journal articles and directly from the authors where available. Using the RoB2 algorithm, studies were assessed with respect to the randomization process (domain 1), bias arising from period and carryover effects (domain S), deviations from the intended interventions (domain 2), missing outcome data (domain 3), measurement of the outcome (domain 4), and selection of the reported results (domain 5). Each domain was categorized as low risk, some concerns or high risk of bias and subsequently an overall risk category was given for each study.

### Meta-analysis

To be included in the meta-analysis studies were required to assess a postural control measure coincident to nGVS as well as a sham or no nGVS condition. Data were extracted for the primary outcome measure that best identified postural control. Only one outcome measure was included from each study. As the studies differed in the reported outcome measures, a standardized mean difference (SMD) along with its standard error (SE) was estimated for each study using Hedges' g formula for repeated measures (Borenstein et al., 2009). The SMD and SE calculation was derived from means, standard deviations, standard errors and 95% confidence intervals (CIs) reported by the studies. The SMD represented the treatment effect of active nGVS compared to sham nGVS or no nGVS on postural control of people with BVP. The SMDs were pooled using a fixed effects meta-analysis model using the metafor package version 3.4 in the R environment for statistical computing (Viechtbauer, 2010). A fixed effects model rather than a random effects model was used as the Cochrane test of heterogeneity suggested a non-significant heterogeneity at a type-I error rate of 5%. Heterogeneity across studies was also reported using the I^2^ statistic. The pooled SMD was considered statistically significant if its 95% confidence interval did not cross zero.

## Results

**Figure 1:**
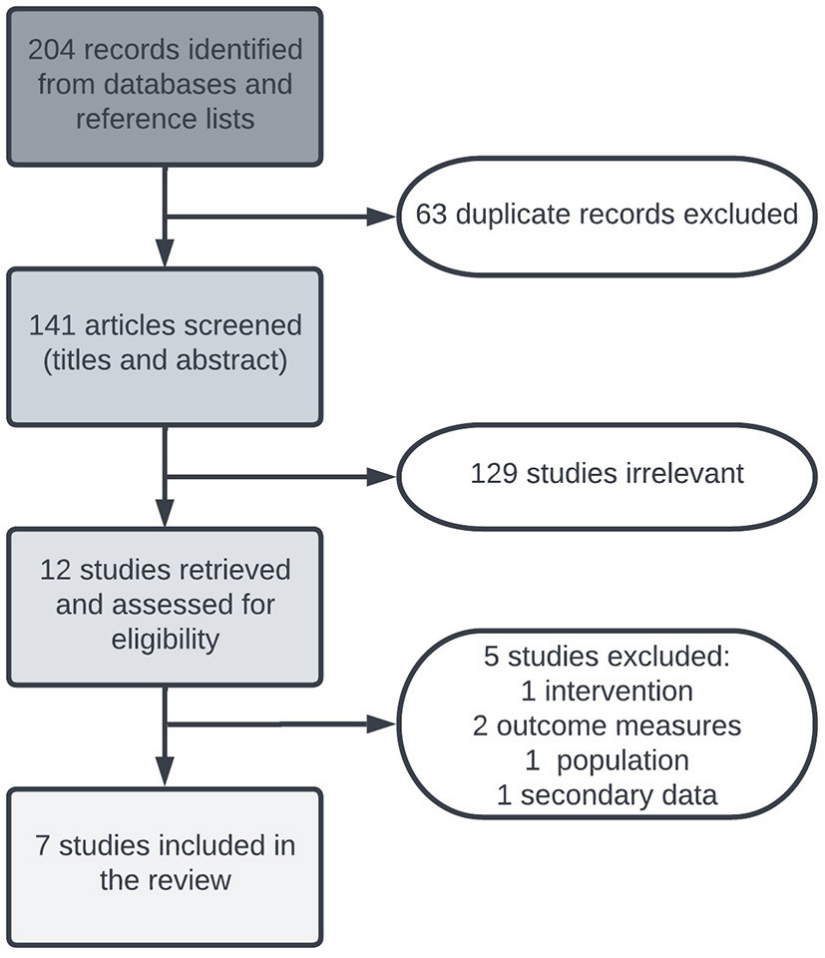
PRISMA diagram of study selection process.

After duplicates were removed the search yielded a total of 141 articles. After the title and abstract screening, 129 were removed as irrelevant and 12 articles went forward for full text review. Five articles were excluded at this stage and seven were retained for data extraction (Figure 1).

### Description of included studies

A total of seven studies were included in this systematic review involving 112 participants with BVP. All participants met the Bárány Society criteria for either full or probable BVP (Table 2) (Strupp et al., 2017). For 66 of the 112 participants the underlying pathology was reported (Table 3), for the others it was not.

**Table 2:** Vestibular diagnostic inclusion criteria used by studies in the systematic review.

**Author ID**	**Subjective report**	**Calorics**	**HIT**	**vHIT**	**MRI**	**Meets Barany criteria**
Barany criteria	Unsteadiness when walking or standing plus at least one of:	Sum of peak SPV < 6 deg/s	–	Bilateral VOR gain < 0.6	–	
	Movement induced oscillopsia or blurred vision					
	Worsening of unsteadiness in darkness and/or on uneven ground					
	No symptoms in static conditions					
	above					
Probable Barany criteria	As for Barany criteria	–	Corrective saccades	–	–	
Chen et al. (2021)	Self-reported history suggestive of BVP	Sum of peak slow phase velocity < 20 deg/s for both ears combined ^*^	Corrective saccades	–	–	Probable
Eder et al. (2022)	–	Sum of mean Peak SPV < 6 deg/sec for each ear^*^	–	Bilateral VOR gain < 0.6	–	Yes
Fujimoto et al. (2018)	–	Peak SPV < 10 deg/s (ice water)	Corrective saccades	–	–	Probable
Iwasaki et al. (2014)	–	Peak SPV < 10 deg/s (ice water)	Corrective saccades	–	–	Probable
Iwasaki et al. (2018)	–	Peak SPV < 10 deg/s (ice water)	Corrective saccades	–	–	Probable
Sprenger et al. (2020)	Reported dizziness, gait unsteadiness and oscillopsia during locomotion and head movements.	Mean peak SPV < 5 deg/s on both sides^*^	Corrective saccades	Bilateral_VOR gain < 0.7	Normal MRI	Probable
Wuehr et al. (2016a)	–	Sum of peak SPV < 10 deg/s for each ear^*^	Corrective saccades	–	–	Probable

HIT, head impulse test; vHIT, video head impulse test; MRI, Magnetic resonance imaging; BVP, bilateral vestibulopathy; SPV, slow phase velocity; VOR, vestibulo ocular refle; –, no data available.

* Based on bithermal protocol.

**Table 3:** Overview of participant's diagnosis.

**Condition**	***n***	**Percentage of participants with a diagnosis**
Not specified	27	–
Unknown	19	–
Idiopathic	35	53
Aminoglycoside toxicity	9	14
Auto immune	6	9
Mitochondrial mutation	5	8
Bilateral Meniere's disease	4	6
Labyrinthitis	3	5
Bilateral vestibular neuritis	1	1.5
Meningitis	1	1.5
Vestibular schwannoma	1	1.5
Trauma	1	1.5

### Study design

**Figure 2:**
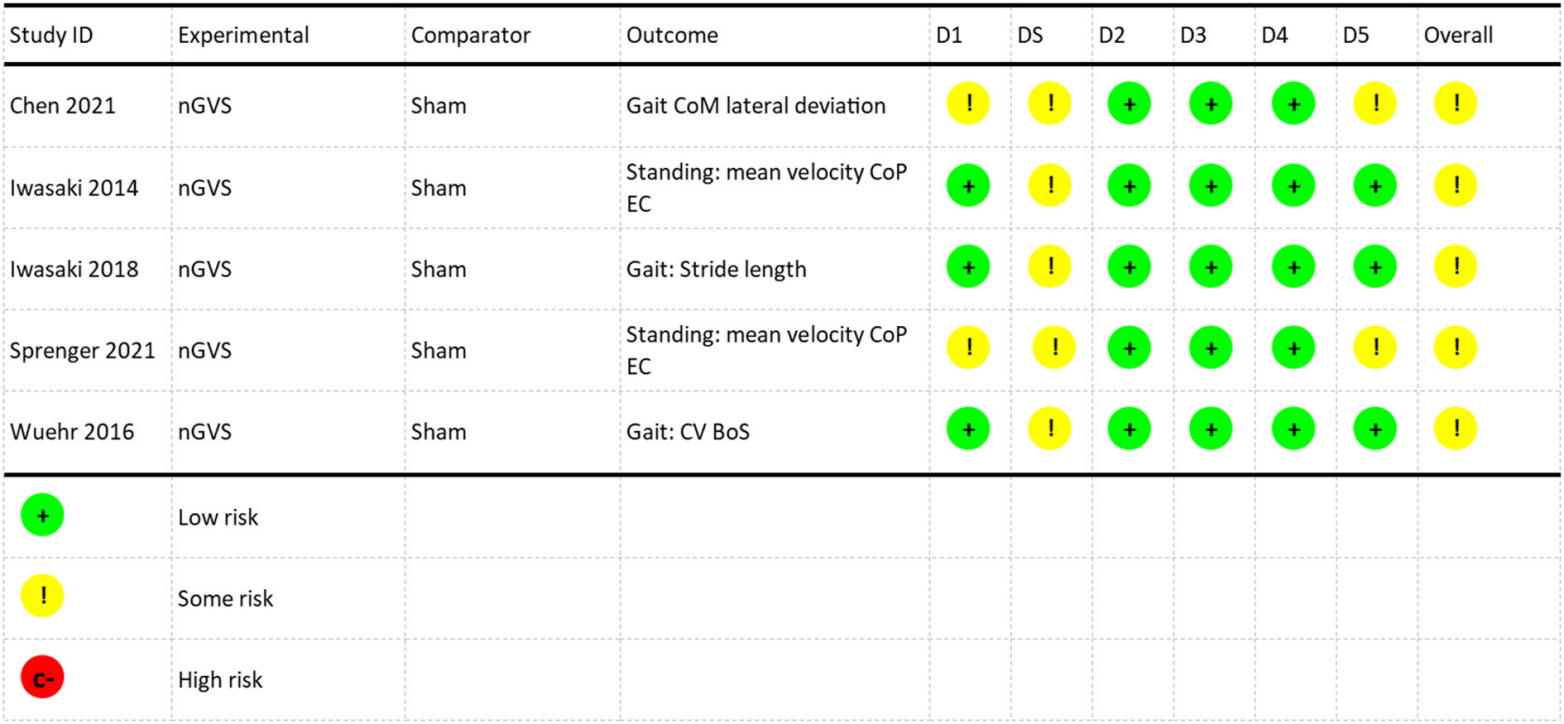
Risk of bias assessment. D1: Randomization Process, DS: Bias arising from period and carryover effects, D2: Deviations from the intended interventions, D3: missing outcome data, D4: Measurement of the outcome, D5: Selection of the reported results. NGVS, Noisy galvanic vestibular stimulation; CoM, center of mass; CoP, center of pressure; EC, eyes closed; CV, coefficient of variation; BoS, base of support.

Studies identified in this review are summarized in Table 4. Five studies used a crossover design and assessed the coincident effect of nGVS on postural control (Iwasaki et al., 2014, 2018; Wuehr et al., 2016a; Sprenger et al., 2020; Chen et al., 2021). The meta-analysis was performed on these five studies. One study used a pre/post experimental design and assessed the post-stimulation effects of nGVS on postural control (Fujimoto et al., 2018) and one was a randomized controlled pilot study (Eder et al., 2022). Six studies explored the efficacy of a single session of nGVS (Iwasaki et al., 2014, 2018; Wuehr et al., 2016a; Fujimoto et al., 2018; Sprenger et al., 2020; Chen et al., 2021). One study evaluated the cumulative effect of multiple sessions of nGVS combined with vestibular rehabilitation (Eder et al., 2022). All studies utilized some method of determining the optimum amplitude of nGVS (Table 4). The methodological quality of the crossover studies used to generate the meta-analysis demonstrated a range from no risk to some risk of bias over the six domains, with all studies categorized overall by the Cochrane RoB2 algorithm as having some risk of bias (Figure 2). The primary risk noted in studies was related to the risk of bias arising from period effects in the Cochrane RoB2 (domain S).

**Table 4:** Summary of study design and outcome measures.

**References**	***n***	**Objective**	**Design**	**Optimization**	**Task**	**Outcome measures**	**Powered for...**.
Chen et al. (2021)	*n* = 10 BVP *n* = 16 healthy	To determine the effect of nGVS on postural stability during gait	Crossover	Lowest RMS sway velocity	Walking EO/EC, Walking +head rotation EO/ EC	Gait: **Lateral deviation of CoM**, gait speed, step length, step width SD of step length and step width. chest-pelvis ratio	80% statistical power *n* = 10 (base of support).
Eder et al. (2022)	*n* = 23 BVP	To examine the synergistic effects of nGVS when combined with standardized vestibular rehabilitation training.	RCT	Greatest improvement—mean velocity, area, and RMS of sway	Standing on foam EC	Standing: **Mean sway velocity EC on foam**. Berg balance scale. Gait: Walking EC-velocity, BoS, CV of stride time, FGA, TuG Functional: DHI, IPAQ, FES-I, ABC	Not specified
Fujimoto et al. (2018)	*n* = 13	To investigate whether long term nGVS continues to improve body balance after cessation of the stimulus in BVP patients.	Pre-post	Greatest improvement—mean velocity, area, and RMS of sway	Standing firm surface EC	Standing: **Mean velocity CoP, area CoP, RMS CoP displacement**, power spectrum of CoP acceleration, power spectral density of CoP- AP and ML	90% statistical power *n* = 13 (mean velocity)
Iwasaki et al. (2014)	*n* = 11 BVP *n*= 21 healthy	To examine the effect of an imperceptible level of nGVS on postural performance in healthy subjects and people with BVP	Crossover	Greatest improvement- mean velocity, area, and RMS of sway	BVP = Standing EC, healthy = Standing EC on foam	Standing: **Mean velocity, area, RMS sway**.	Not specified
Iwasaki et al. (2018)	*n* = 12 BVP *n* =19 healthy	To examine the effect of an imperceptible level of nGVS on dynamic locomotion in normal subjects as well as patients with bilateral vestibulopathy	Crossover	Highest gait velocity	Gait EO preferred speed	Gait: **velocity, stride length, stride time**, lateral movement distance, vertical movement distance, CV stride time, CV lateral movement distance, CV vertical movement distance.	Not specified
Sprenger et al. (2020)	*n* = 30 BVP *n* = 24 Healthy	Does nGVS improve postural control in comparison to sham stimulus in context dependent conditions.	Crossover	80% perceptual motion threshold (1 Hz sinusoidal GVS)	Standing firm surface (EO EC), standing foam (EO EC), standing firm surface dual task (EO EC) ± nGVS	Standing: **CoP mean velocity**	Powered at 80% *n* = 24
Wuehr et al. (2016a)	*n* = 13	Examine the effect of imperceivable levels of nGVS on walking performance in patients with BVP.	Crossover	80% cutaneous sensory threshold	Gait EO preferred, slow (25%) and fast (125%) speed ± nGVS	Gait: **Stride time, stride length, BoS, double support phase**. CV stride time, stride length, BoS and bilateral phase synchronization.	Not specified

BVP, bilateral vestibulopathy; nGVS, noisy galvanic vestibular stimulation; RMS, root mean squared; EO, eyes open; EC, eyes closed, CoM, center of mass; BoS, base of support; FGA, functional gait analysis; TUG, timed up and go; DHI, dizziness handicap inventory, IPAQ, international physical activity questionnaire; FES-I, falls efficacy scale international; ABC, Activities Balance Confidence scale; CV, coefficient of variation.

Bold—primary outcome measure.

### Effect of nGVS on standing balance

**Figure 3:**
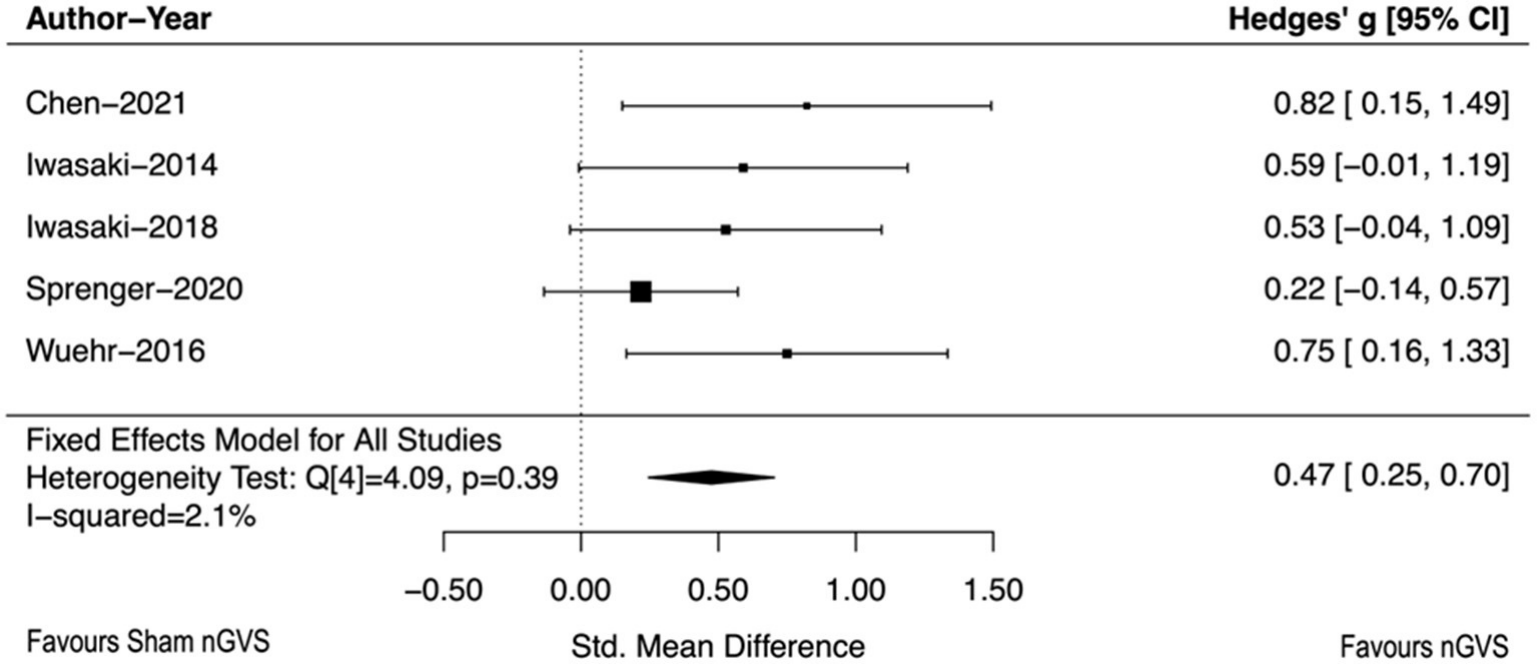
Forest plot of the immediate effect of nGVS on postural control.

Meta-analysis of the five studies assessing the coincident effect of nGVS revealed a moderate effect in favor of nGVS improving postural control during standing and walking (pooled SMD = 0.47 95% CI, [0.25,0.07]) (Figure 3) (Brydges, 2019). There was non-significant heterogeneity in the sample (*I*2 = 2.1%, *p* = 0.39).

The coincident effect of nGVS on postural control was evaluated with nGVS actively stimulating the participant during the measurement period. The post-stimulation effect of nGVS on postural control was measured after active nGVS ceased, with the period described as time since the nGVS current stopped.

#### Standing with vision available

One study (Sprenger et al., 2020) investigated the coincident effect of nGVS on sway velocity whilst standing with eyes open on a firm surface and on foam. They found no significant effect of nGVS on postural control (Table 5).

**Table 5:** nGVS effect standing with eyes open.

**Reference**	**Condition**	**Surface**	**Period**	**NR**	**Sig**	**Mean velocity CoP baseline (cm/s)**	**Mean velocity CoP nGVS (cm/s)**
Sprenger et al. (2020)	BVP	Firm	During	1	NS	1.63 ± 0.08	1.63 ± 0.08
	BVP low threshold	Firm	During	0.98	NS	1.48 ± 0.18	1.45 ± 0.10
	BVP high threshold	Firm	During	1.01	NS	1.80 ± 0.20	1.83 ± 0.18
	BVP	Foam	During	0.89	NS	2.41 ± 0.49	2.15 ± 0.14
	BVP low threshold	Foam	During	0.84	NS	2.57 ± 0.53	2.16 ± 0.18
	BVP high threshold	Foam	During	0.94	NS	2.25 ± 0.18	2.14 ± 0.29

NR, Normalized ratio; Sig, Significance; CoP, Center of Pressure; nGVS, noisy galvanic vestibular stimulation; BVP, bilateral vestibulopathy; NS, not significant; low threshold, group of participants with BVP who perceived motion during galvanic vestibular stimulation within the same range of amplitudes as healthy participants; high threshold, group of participants with BVP who perceived motion during galvanic vestibular stimulation only at a higher amplitude than healthy participants.

#### Standing with vision eliminated

Three studies investigated nGVS in standing with vision eliminated on a firm surface (Table 6) (Iwasaki et al., 2014; Fujimoto et al., 2018; Sprenger et al., 2020). They found an immediate reduction in sway velocity with coincident nGVS of 1.19 cm/s (normalized ratio (NR) 0.68, *p* < 0.001) (Iwasaki et al., 2014) and 1.52 cm/s (NR 0.75, *p* < 0.006) (Sprenger et al., 2020) with a post- stimulation effect lasting for 3 h post-stimulation (NR 0.77, *p* < 0.01 at zero mins post stimulation and NR 0.85, *p* < 0.001 at 3 h post stimulation) (Fujimoto et al., 2018). Sprenger et al. (2020) noted the benefit appeared to be weighted toward BVP participants with higher motion perception thresholds when stimulated with galvanic vestibular stimulation (NR 0.75, *p* < 0.006) compared to participants with BVP who had a motion perception threshold within the normal range (NR 0.92, *p* > 0.05). In the only trial that measured standing on a foam surface with eyes closed, Sprenger et al. (2020) reported that during stimulation nGVS did not have a significant effect on mean sway velocity in this condition (Table 7).

**Table 6:** nGVS effect standing with eyes closed on firm surface.

**References**	**Population**	**Period**	**NR**	**Significance**	**Mean velocity CoP sham 0 mA nGVS (cm/s)**	**Mean velocity CoP optimum nGVS (cm/s)**
Iwasaki et al. (2014)	BVP	During	0.68	*p* < 0.001	3.69 ± 0.65	2.50 ± 0.35
Sprenger et al. (2020)	BVP	During	0.81	NS	4.02 ± 0.48	3.24 ± 0.74
	BVP low threshold	During	0.92	NS	2.40 ± 1.9	2.21 ± 0.17
	BVP high threshold	During	0.75	*p* < 0.006	6.18 ± 1.58	4.66 ± 1.04
Fujimoto et al. (2018)	BVP	0 mins	0.77	*p* < 0.01	–	–
	BVP	30 min	0.83	*p* < 0.01	–	–
	BVP	1 h	0.84	*p* < 0.05	–	–
	BVP	2 h	0.83	*p* < 0.01	–	–
	BVP	3 h	0.85	*p* < 0.001	–	–
	BVP	4 h	0.94	NS	–	–
	BVP	5 h	0.96	NS	–	–
	BVP	6 h	0.96	NS	–	–

NR, normalized ratio; CoP, center of pressure; nGVS, noisy galvanic vestibular stimulation; BVP, bilateral vestibulopathy; EC, eyes closed; NS, nonsignificant result; –, no data available; low threshold, group of participants with BVP who perceived motion during galvanic vestibular stimulation within the same range of amplitudes as healthy participants; high threshold, group of participants with BVP who perceived motion during galvanic vestibular stimulation only at a higher amplitude than healthy participants.

**Table 7:** nGVS effect standing with eyes closed on foam.

**References**	**Condition**	**NR**	**Significance**	**Mean velocity CoP Sham nGVS 0 mA (cm/s)**	**Mean velocity CoP optimum nGVS (cm/s)**
Sprenger et al. (2020)	BVP	1.17	NS	8.45 ± 1.08	9.89 ± 0.97
	BVP low threshold	1.18	NS	7.70 ± 0.92	9.15 ± 1.22
	BVP high threshold	1.14	NS	9.34 ± 0.98	10.66 ± 1.56

NR, normalized ratio; CoP, center of pressure; nGVS, noisy galvanic vestibular stimulation; BVP, bilateral vestibulopathy; NS, non-significant result; low threshold, group of participants with BVP who perceived motion during galvanic vestibular stimulation within the same range of amplitudes as healthy participants; high threshold, group of participants with BVP who perceived motion during galvanic vestibular stimulation only at a higher amplitude than healthy participants.

### Effect of nGVS on gait

#### Spatiotemporal gait parameters

During over-ground walking, gait speed and stride length increased, and stride time decreased with coincident nGVS (Iwasaki et al., 2018). There was no effect during paced treadmill walking (Table 8) (Wuehr et al., 2016a).

**Table 8:** nGVS effect on the spatiotemporal parameters of gait.

**References**	**Gait speed**	**Gait velocity** **(% change)**	**Stride time** **(% change)**	**Stride length** **(% change)**	**BoS** **(% change)**	**Double support** **(% change)**
Wuehr et al. (2016a)	Slow (25% preferred)	–	2.2 NS	1.9 NS	2.0 NS	7.0 NS
Wuehr et al. (2016a)	Preferred	–	1.7 NS	1.5 NS	6.7 NS	2.8 NS
Iwasaki et al. (2018)	Preferred	12.8 ± 1.3	5.8 ± 0.001	8.0 ± 0.01	–	–
		*p* < 0.0001	*p* < 0.0001	*p* < 0.001		
Wuehr et al. (2016a)	Fast (125% preferred)	–	0.9 NS	0.6 NS	3.3 NS	1.1 NS

BoS, base of support; EO, eyes open; NS, not statistically significant; –, not measured.

#### Gait variance measures

Of the three studies that measured the effect of coincident nGVS on gait variance, there was a significantly reduced variability in gait parameters in two of the studies (Table 9). This was particularly evident in base of support and step width measures with a 51.1% reduction in SD of step width (*p* = 0.009) (Chen et al., 2021) and a 13.1% (*p* < 0.05) reduction in the coefficient of variation of base of support (Wuehr et al., 2016a) during walking at the participants' preferred speed. There was no significant change in the coefficient of variation of stride time, except at slow speed (Wuehr et al., 2016a) where it was 32.2% less variable (*p* < 0.05). This reduction in gait variance and improvement in stability was also noted in more complex gait tasks, for example, whilst walking with head turns (Chen et al., 2021).

**Table 9:** nGVS effect on gait variance.

**Author**	**Activity**	**CV stride time** **(% change)**	**CV stride length** **(% change)**	**CV BoS** **(% change)**	**CV phase coordination** **(% change)**	**SD of width** **(% change)**	**Walking deviation** **(% change)**
Wuehr et al. (2016a)	Slow gait (25% preferred) EO	32.2 *p* < 0.05	35.0 *p* < 0.05	7.9 *p* < 0.05	33.1 *p* < 0.05	–	–
Wuehr et al. (2016a)	Preferred gait speed EO	3.0 NS	12.8 *p* < 0.05	13.1 *p* < 0.05	26.2 NS	–	–
Chen et al. (2021)	Preferred gait speed EO	–	–	–	–	51.1 *p* = 0.009	20.6 *p* = 0.037
Iwasaki et al. (2018)	Preferred gait speed EO	29 NS	–	–	–	–	–
Chen et al. (2021)	Preferred gait speed EC	–	–	–	–	38.9 *p* = 0.028	34.6 *p* = 0.017
Wuehr et al. (2016a)	Fast gait (125% preferred) EO	8.3 NS	9.7 NS	3.0 *p* < 0.05	6.1 NS	–	–
Chen et al. (2021)	Preferred gait speed EO + head turns	–	–	–	–	36.9 *p* = 0.028	NS
Chen et al. (2021)	Preferred gait speed EC + had turns	–	–	–	–	52.8 *p* = 0.022	36.6 *p* = 0.047

CV, coefficient of variation; BoS, base of support; SD, standard deviation; EO, eyes open; EC, eyes closed; NS, non-significant result; –, not measured.

### nGVS augmented vestibular rehabilitation

In the only study to report the effects of nGVS-augmented rehabilitation, Eder et al. (2022) reported that 6 sessions of nGVS over 2 weeks together with vestibular rehabilitation did not significantly reduce postural sway in people with BVP. There was no difference in postural control measures between the experimental group receiving nGVS plus vestibular rehabilitation and the control group receiving sham nGVS plus vestibular rehabilitation (Eder et al., 2022).

## Discussion

As the first systematic review and meta-analysis of the effectiveness of nGVS in people with BVP, this work advances the understanding of this modality and the contexts where nGVS may have the most efficacy. Meta-analysis demonstrated that the coincident effect of nGVS on postural stability in people with BVP was to improve balance. The very low heterogeneity of the results indicates that the results across studies were consistent, with little variability. All sampled studies favored nGVS with consistent effect sizes (>0.5), excepting one study which reported a smaller effect size (0.22).

### Effect of pathology, location, and severity on efficacy of nGVS

While the neurophysiological basis of nGVS is not fully understood, it seems likely that the extent of peripheral vestibular integrity and baseline postural stability makes a difference to a person's responsiveness to nGVS (Fujimoto et al., 2019a; Herssens and McCrum, 2019; Lajoie et al., 2021). Evidence points to nGVS being most effective in people with suboptimum vestibular function, yet at the same time some intact vestibular hair cells and nerve afferents are required to enable a vestibular afferent signal to be transmitted and reach the CNS (Dlugaiczyk et al., 2019; Lajoie et al., 2021). Clinical evidence supports this assertion. nGVS was not effective in people with complete bilateral loss (Schniepp et al., 2019), whereas people with incomplete vestibular loss exhibited a significant improvement in postural control with nGVS compared to those with normal vestibular function (Nooristani et al., 2021).

The Barány Society's criteria for the diagnosis of BVP is based on horizontal semicircular canal function. Consequently, the significance of residual function from the remaining vestibular organs in determining responsiveness to nGVS is unclear. Two studies in people with BVP included individual patient data on saccular function based on raw cervical vestibular evoked myogenic potential (cVEMP) amplitudes (Wuehr et al., 2016a; Iwasaki et al., 2018). cVEMP amplitudes represent modulation of motor potentials recorded from the sternocleidomastoid muscle in response to acoustic stimulation of the saccule and are used to illustrate the integrity of the sacculocollic neural pathway (Colebatch and Rothwell, 2004). No significant association has been found between saccular function and responsiveness to nGVS to date (Wuehr et al., 2016a). However, the nuances around residual function of the vestibular system require further investigation, in particular, the role of high frequency vestibular afferents (Bae et al., 2021) and otolith function (Keywan et al., 2019) in the potential for restoration of postural control. Due to the rarity of BVP, we encourage more authors to make supplementary files available, with deidentified information regarding individual vestibular assessment results and the participants' response to nGVS, in order to contribute to the identification of participants for whom nGVS is most likely to be effective in the future.

### Standing balance

#### Coincident effects of nGVS on standing postural control

nGVS had no significant effect on postural control when people stood with their eyes open (Table 9), regardless of proprioceptive afferent input (i.e., standing on a firm surface or standing on foam) (Sprenger et al., 2020). In contrast, older adults without BVP had a significant reduction in mean sway velocity standing with eyes open (*p* = 0.005) (Inukai et al., 2018a), and in studies of healthy young people the results have been mixed: showing benefits with nGVS for some but not all participants (Inukai et al., 2020a,b; Matsugi et al., 2020). In people with BVP it is likely that there is longstanding CNS reweighting of the balance response toward utilization of visual afferent input when vision is available (Medendorp et al., 2018; Helmchen et al., 2019). CNS reweighting to adapt to the facilitated vestibular input by nGVS is likely to require longer than a 30 s exposure. This may contribute to the lack of immediate benefit from nGVS augmented vestibular input in people with BVP. Conversely, older adults have unavoidable deterioration in vision due to age related changes to the anatomy of the eye, reducing the quality of visual input to the CNS (Saftari and Kwon, 2018). Whilst vision is still considered a critical component of the older adult balance response (Saftari and Kwon, 2018), adaptive changes to the CNS reweighting of afferent inputs may predispose them to having greater sensitivity to vestibular augmentation.

Two studies investigating the balance response of people with BVP standing on a firm surface with eyes closed demonstrated a significant reduction in sway velocity (Iwasaki et al., 2014, 2018; Sprenger et al., 2020). In the absence of vision, reweighting of the postural control mechanism to increase reliance on vestibular and proprioceptive afferent information occurs (Asslander and Peterka, 2016). Accordingly, once the visual system was removed from the balance response, augmentation of the vestibular pathway appears to have benefit. Sprenger et al. (2020) found that this response was specific to people in their high threshold group. The high threshold group required higher amplitude GVS stimulation to reach vestibular firing threshold (as determined by perception of motion in response to galvanic stimulation). This group also had higher baseline sway velocity, suggesting greater initial postural instability. These findings are consistent with conclusions drawn in other studies. Nooristani et al. (2021) found older adults with vestibular impairment showed more responsiveness to nGVS stimulation than those with intact vestibular systems, and Inukai et al. (2018a,b); Inukai et al. (2020c) found nGVS was more effective at improving balance in people who present with some baseline postural instability.

Once balance is heavily weighted to be reliant on the vestibular system by removing vision and reducing proprioceptive information (standing on foam with their eyes closed), postural sway has high variability in all populations (Rashid et al., 2021). In the one study that investigated people with BVP standing on foam with their eyes closed nGVS did not have a significant effect on sway velocity (Sprenger et al., 2020). This pattern has also been seen in healthy young adults (Inukai et al., 2020a,b; Matsugi et al., 2020; Asslander et al., 2021), and while overall Mulavara et al. (2011) and Goel et al. (2015) concluded nGVS improved postural stability, when we look at individual participant results, only approximately half the participants in these two studies had reduced sway velocity standing on foam with their eyes closed. While it seems intuitive that augmenting the vestibular system should improve balance when the task is dependent on vestibular inputs, there are several reasons why we may not see the anticipated improvement in healthy populations or in people with BVP. Firstly, standing on a compliant surface is a complex postural task influenced not only by sensory information but also by the task, environment, and context (Shumway-Cook and Woollacott, 2007). The vestibular system provides information on position and movement of the head with respect to gravity, however, without the context of visual or somatosensory information, the vestibular system cannot distinguish the importance and relevance of postural change (Bayot et al., 2018). Therefore, even with facilitation to bring the vestibular signal up to threshold, in the absence of proprioception and vision, the vestibular system may not be sufficiently sensitive to maintain stability. Secondly, measuring velocity of the center of pressure is not a true record of body sway but more accurately measures the activity of the motor system moving the center of pressure (Ruhe et al., 2011). Therefore, a task such as standing on foam with eyes closed, where maintaining the center of mass within the base of support requires extensive exertion of the muscles around the ankles, may reduce the sensitivity of this measure as an indication of postural sway (Matsugi et al., 2020). This is a thought-provoking area for further research that will help us understand the potential for nGVS as a treatment modality in more complex multifactorial tasks with diminished afferent sensory information.

#### Lasting effects of nGVS on postural control

The sustained effects of nGVS on postural control are not yet clear. Fujimoto et al. (2018) investigated the sustained effects of nGVS on people with BVP, finding that 30 mins of stimulation significantly reduced sway velocity for 3 h after nGVS was removed. These findings are comparable to those found in older adults where a significant effect on postural sway was evident for up to 4 h after stimulation with a second burst of 30 mins nGVS sustaining this effect for a further 4 h (Fujimoto et al., 2016). However, these studies have been criticized for the absence of a comparative control group using sham stimulation, as the use of repeated measures creates risk of a learning effect that may influence the results. Nooristani et al. (2019b) investigated post-stimulation effects of nGVS in healthy people, using a comparative sham group. They found a significant reduction in sway velocity immediately after, and at 1 h post stimulation, in both the nGVS and sham nGVS groups with no significant difference between groups. Similarly, in a sham-controlled crossover study Keywan et al. (2020) found no significant effect on motion perception immediately after, or 30 mins subsequent to nGVS or sham nGVS. These findings support the concerns of bias in earlier studies. Further investigations using a sham stimulation control group will help clarify any continued effects of stimulation.

### Effect of nGVS on gait

#### Spatiotemporal parameters of gait

Two studies have investigated the effect of nGVS on the spatiotemporal parameters of gait in people with BVP. Iwasaki et al. (2018) found that nGVS increased gait speed and stride length, and decreased stride time, in people with BVP during overground walking. A similar effect was found in healthy young people during overground walking and walking on unsteady surfaces (Piccolo et al., 2020). To the contrary, Wuehr et al. (2016a,b) found there was no change in spatiotemporal gait parameters in healthy or BVP populations during walking at slow, preferred, and fast speeds. However, because their studies were on a treadmill and gait speed was predetermined, the influence of the treadmill pacing may have had an overriding influence (Wuehr et al., 2016a,b). Further research is required to determine whether gait speed and its associated parameters are affected by nGVS.

Three studies have reported on gait variance measures in people with BVP, with studies reporting significantly reduced gait variability particularly in step width and base of support, the lateral components of gait stability (Wuehr et al., 2016a; Iwasaki et al., 2018; Chen et al., 2021). This finding is noteworthy, as lateral gait variance has been cited as a defining feature of gait imbalance in people with BVP (Schniepp et al., 2019). Decreased step width variability (Wuehr et al., 2016b; Piccolo et al., 2020) and improved stability during lateral perturbation of the support surface (Mulavara et al., 2015) have also been found in nGVS studies on young healthy people, strengthening support for the role of nGVS to augment vestibular afferents and maintain stable gait (Schniepp et al., 2019).

#### Gait with head turns

The vestibular system has a critical role stabilizing vision *via* the vestibulo-ocular and vestibulospinal reflexes. To this end researchers have looked at gait with head turns to challenge the vestibular system during gait. This task is a demanding one for both healthy people and people with BVP (Ko et al., 2020). Ko et al. (2020) assessed coherence to 2 Hz turning during gait and discovered significantly improved coherence to 2 Hz head turns during gait with nGVS in people with BVP (0.35 ± 0.24 Hz without nGVS, 0.46 ± 0.28 Hz with nGVS), and healthy young people (*p* = 0.018). Nevertheless, despite the challenge of gait with head turns, Chen et al. (2021) found a significant reduction in the standard deviation of stride width walking with head turns with both eyes open and closed, suggesting that nGVS can offer increased stability during gait even during a more challenging task. They also found reduced gait deviation during gait with head turns when the eyes were closed, but not when the eyes were open. This supports the visual system's dominant role in the control of gait trajectory when vision is available. Further research into gait in complex situations will help us understand more about the vestibular contribution to stability and how nGVS may influence mobility in real life situations which are innately more complex and unpredictable than laboratory-based investigations.

Overall, there is limited research into the effect of nGVS on gait. However, research to date has identified the potential of nGVS to improve gait stability in people with BVP even during challenging tasks. This is particularly relevant, as gait stability is one of the primary long term deficits affecting people with BVP (Wuehr et al., 2022a).

### Effect of nGVS over the longer term

While there is good evidence for the immediate benefit of nGVS to improve postural stability during standing and walking with some residual effect following the removal of the stimulation, there has been speculation about the effect of nGVS as an adjunct to rehabilitation (Iwasaki et al., 2014; Chen et al., 2021; Lotfi et al., 2021). Eder et al. (2022) piloted nGVS as an adjunct to vestibular rehabilitation in people with BVP for 30 mins three times a week for 2 weeks. They found that vestibular rehabilitation for 2 weeks yielded moderate improvements in functional mobility as measured by the timed up and go, and functional gait analysis, but found no significant difference between the nGVS and sham group on any outcome measures. There were no reports of dropouts or adverse events in this study, suggesting this higher dose of nGVS spread over weeks is feasible and acceptable. Future research would benefit from investigating augmentation of vestibular rehabilitation over a longer duration, more in line with clinical practice (Hall et al., 2021). Ensuring the outcome measures used have been demonstrated to be sensitive to nGVS in people with BVP may also provide a more accurate representation of any effect.

### Limitations

While research into nGVS as a modality to improve balance has been investigated for several decades now, there remains limited high-quality research in people with BVP, and notably, a lack of research including a comparative control group (Nooristani et al., 2019b; Sprenger et al., 2020). Future research will benefit from inclusion of individual participants' vestibular assessment results and responsiveness to nGVS, helping build our understanding of who is most likely to respond to nGVS.

While there is acknowledgment in the literature that the parameters of the nGVS waveform are important (Dlugaiczyk et al., 2019; Fujimoto et al., 2019b; Herssens and McCrum, 2019; Stefani et al., 2020; Lajoie et al., 2021), the limited systematic evaluation of parameter settings, and variability in individual's response to nGVS make it unclear how the choice of stimulation parameters may affect responsiveness and outcomes. Within the studies evaluated in this review some of the contradictory results of the effect of nGVS on postural stability may be related to the different methods utilized to optimize the parameters. In the five studies used for meta-analysis, current amplitude was determined by four different means; postural sway velocity (Iwasaki et al., 2014; Chen et al., 2021), cutaneous sensory threshold (Wuehr et al., 2016a), motion perception threshold (Sprenger et al., 2020) and gait velocity (Iwasaki et al., 2018). Further understanding of the science underpinning the choice of nGVS parameters and how to determine their optimal settings is required to develop this work.

As much of the initial nGVS research was exploratory there are some methodological issues that are only now being redressed. The research upon which our theoretical underpinnings are based has been primarily performed in young healthy adults whom we now understand respond differently to nGVS than those with higher postural sway or vestibular impairment (Fujimoto et al., 2019a; Nooristani et al., 2021). These initial studies have often looked at multiple intensities, yet only reported on the intensity related to optimal performance (Goel et al., 2015; Mulavara et al., 2015; Fujimoto et al., 2016; Iwasaki et al., 2018) or have examined one intensity compared to sham stimulation (Inukai et al., 2018a,b, 2020a,b,c; Nooristani et al., 2019a,b; Matsugi et al., 2020). Research investigating performance alterations across a broad spectrum of stimulation intensities to account for the presence or absence of stimulation-induced stochastic resonance is starting to fill these gaps in our knowledge (Galvan-Garza et al., 2018; Asslander et al., 2021; Wuehr et al., 2022b). Investigation of parameter induced patterns of performance across a range of frequencies and amplitudes in people with reduced vestibular function or high baseline postural sway is required to advance our understanding of nGVS.

## Conclusions

The meta-analysis demonstrated that nGVS has a moderate coincident effect to improve postural stability in people with BVP. However, these improvements are context specific. Where the visual system can dominate, enhancing the vestibular system does not improve postural stability. When the visual input is eliminated but there is sufficient somatosensory afferent feedback, nGVS reduces sway velocity improving balance, particularly in the unsteady; those with high baseline sway. When visual input and somatosensory input are removed (standing on foam with eyes closed), augmenting the vestibular system has no effect on postural control. During gait, even challenging gait with eyes closed and head turns, nGVS improved stability by reducing gait variability. Overall, these findings support further research into the use of nGVS over longer periods with the potential to use this stimulation as an orthotic.

Preliminary research into using nGVS as an adjunct to vestibular rehabilitation suggests it is a feasible treatment. However, further research is required with treatment over a longer duration, using outcome measures that have been found to be sensitive to vestibular rehabilitation and nGVS before we can draw any reliable conclusions about its efficacy to augment rehabilitation.

## Data availability statement

The original contributions presented in the study are included in the article/supplementary materials, further inquiries can be directed to the corresponding author.

## Author contributions

RM and DT conceptualized the study. RM performed the literature search, extracted and critically reviewed the data, and wrote the first draft of the manuscript. RM and SR independently reviewed the articles with help from DT. UR performed the statistical analysis. PS, RT, and DT provided feedback at all stages of manuscript development. All authors reviewed and approved the final manuscript.

## Funding

This study was supported by the Health Research Council of New Zealand, Grant Numbers: HRC22/363 and HRC19/632.

## Conflict of interest

The authors declare that the research was conducted in the absence of any commercial or financial relationships that could be construed as a potential conflict of interest.

## Publisher's note

All claims expressed in this article are solely those of the authors and do not necessarily represent those of their affiliated organizations, or those of the publisher, the editors and the reviewers. Any product that may be evaluated in this article, or claim that may be made by its manufacturer, is not guaranteed or endorsed by the publisher.
